# Rethinking access to care: A spatial-economic analysis of the potential impact of pharmacy closures in the United States

**DOI:** 10.1371/journal.pone.0289284

**Published:** 2023-07-27

**Authors:** Omolola E. Adepoju, Amin Kiaghadi, Darya Shokouhi Niaki, Adebosola Karunwi, Hua Chen, LeChauncy Woodard

**Affiliations:** 1 University of Houston College of Medicine, Houston, Texas, United States of America; 2 Humana Integrated Health Systems Sciences Institute, Houston, Texas, United States of America; 3 University of Houston Department of Civil and Environmental Engineering, Houston, Texas, United States of America; 4 Virginia Commonwealth University Department of Biostatistics, Richmond, Virginia, United States of America; 5 University of Houston College of Pharmacy, Houston, Texas, United States of America; University of Washington, UNITED STATES

## Abstract

Data chronicling the geo-locations of all 61,589 pharmacies in the U.S. (from the Homeland Infrastructure Foundation-Level Data (HIFLD) Open Data interface, updated on April 2018) across 215,836 census block groups were combined with Medically Underserved Areas (MUAs) information, and the Centers for Disease Control and Prevention’s Social Vulnerability Index (CDC-SVI). Geospatial techniques were applied to calculate the distance between the center of each census block and the nearest pharmacy. We then modeled the expected additional travel distance if the nearest pharmacy to the center of a census block closed and estimated additional travel costs, CO2 emissions, and lost labor productivity costs associated with the additional travel. Our findings revealed that MUA residents have almost two times greater travel distances to pharmacies than non-MUAs (4,269 m (2.65 mi) vs. 2,388 m (1.48 mi)), and this disparity is exaggerated with pharmacy closures (107% increase in travel distance in MUAs vs. 75% increase in travel distance in non-MUAs). Similarly, individuals living in MUAs experience significantly greater average annual economic costs than non-MUAs ($34,834 ± $668 vs. $22,720 ± $326). Our findings suggest the need for additional regulations to ensure populations are not disproportionately affected by these closures and that there is a significant throughput with community stakeholders before any pharmacy decides to close.

## Introduction

Pharmacies play a crucial role in communities by providing a wide range of healthcare services, including medication dispensing, medication management, patient education and counseling, point-of-care testing, and immunizations [1, 2]. Medication dispensing is a central part of preventive treatment and management for various diseases and can potentially delay the progression of preventable healthcare utilization and complications. Patient counseling provided by pharmacists significantly improves compliance with treatment, which, in turn, can reduce overall healthcare costs [3]. Pharmacies also play a critical role in providing preventive services. Prior studies had shown that when pharmacies were used as influenza vaccination sites, there was a 7-week reduction in the time necessary to vaccinate 80% of the population [4]. In particular, since the onset of the COVID-19 pandemic, community pharmacies have played a vital role in public health efforts and in filling the gaps in an over-stressed healthcare system. More than a reliable source of healthcare and COVID-19 information, pharmacists have also been a significant contributor to the widespread distribution of the SARS-CoV-2 vaccine, administering about 200 million doses to the American people [1]. Serving as an integral access point to primary care, pharmacies are often seen as places of potential interactions with licensed healthcare professionals, typically without needing an appointment.

Despite the critical role of pharmacies within the health care system, a significant rise in completed and planned pharmacy closures has occurred throughout the nation [5]. For example, in November 2021, CVS, one of America’s largest pharmacy chains, announced plans to close almost 900 store locations over three years [5, 6]. Impending closures among other pharmacy chains, such as Rite Aid, decreased hours due to staffing shortages, and employee burnout also threaten access to necessary care [7]. One such barrier to healthcare access associated with pharmacy closure is increased patient travel distance and time. In a 2013 study, investigators found that 17% of pharmacy closures in communities with no other healthcare providers led to an average of at least 60 additional minutes of travel time to reach an urbanized area [8]. This finding is amplified in poorer populations living in remote areas [9].

"Pharmacy deserts" [10, 11], which are areas with no or only a few pharmacies, occur most often in primarily Black and Hispanic communities, low-income communities, and regions identified as Medically Underserved Areas (MUAs). As defined by the Health Resources and Services Administration (HRSA), characteristics of MUAs include the lack of primary care providers, an elevated infant mortality rate, a high poverty rate, and/or a high elderly population [12]. The MUA shortage designation allows these areas to participate in certain federal programs and receive additional benefits to improve healthcare access for their residents [13, 14].

Prior work suggests that pharmacies in low-income and vulnerable neighborhoods are at the highest risk of closing. The risk of closure is higher in urban areas among the low-income, uninsured population [5]. With geographic barriers as a primary obstacle to pharmacy access, longer travel distance to the next pharmacy may significantly lower access to primary care [5]. As a result, these closures may detrimentally affect the health outcomes of acute and chronic disease patients and impose a significant burden on the U.S. healthcare system. Furthermore, for medically underserved rural and urban residents, who already experience more significant barriers to pharmacy and healthcare access, closures may exacerbate such health disparities.

Given the anticipated inequitable impact of pharmacy closures on already underserved communities, a quantitative assessment (i.e., descriptive research) of the potential outcomes associated with such closures is essential for resource allocation and may have important policy implications for future closings. This study examines the expected effects of pharmacy closures, regardless of brand pharmacy type, by modeling the expected additional travel distance if the nearest pharmacy to the center of a census block group (as a proxy to the households) was closed and the economic ramifications associated with this increase. This approach allows a comparison of the additional burdens in areas of higher social vulnerability, as captured by the Centers for Disease Control and Prevention’s Social Vulnerability Index (CDC-SVI) and MUA status. Studies of this nature can shed light on the relationship between pharmacies and access to care issues and may provide crucial information that ensures disparities in vulnerable neighborhoods do not worsen due to pharmacy closures.

## Materials and methods

### Data acquisition

**Fig 1:**
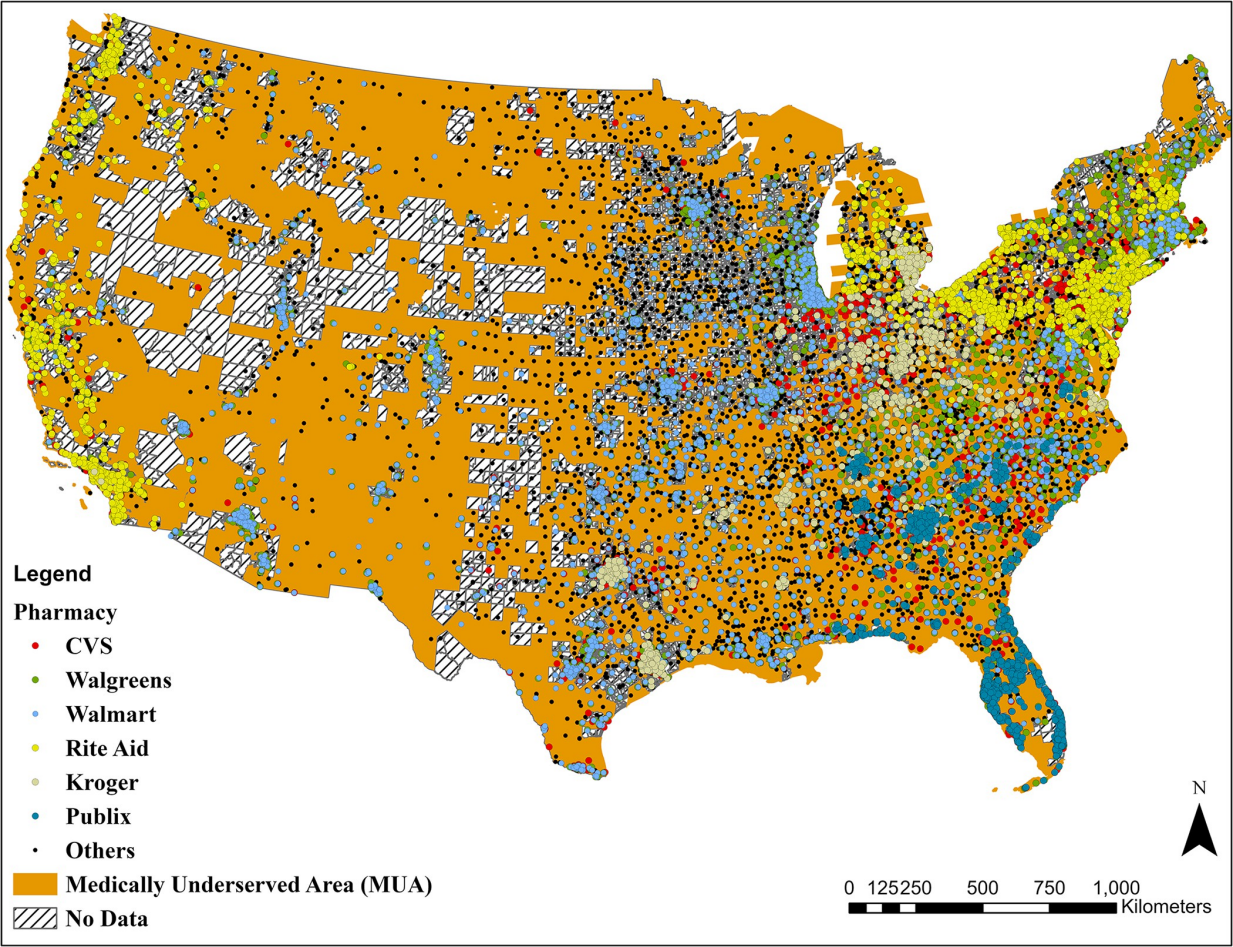
Spatial distribution of pharmacies (classified by the major brands, n = 61,589) overlayed by the locations of Medically Underserved Areas (MUAs).

Four data sources were combined: 1) Census block groups data from the U.S. Census (with an average area and population of 36 km^2^ and 1,483 people, respectively), 2) community/retail pharmacy brand and location data, 3) MUA boundaries, and 4) CDC-SVI estimates. We downloaded U.S. census data at block group spatial resolution and the associated shapefile containing spatial boundaries and areas from the National Historical Geographic Information System (NHGIS) database [15]. ArcMap 10.8.1 [16] was used to limit the data to the contiguous U.S. (using the US contiguous Albers Equal Area Conic projection), which led to a shapefile with a total of 215,836 census block groups. The names and locations of community/retail pharmacies and dialysis centers (the data are provided jointly, updated on January 2022) in the U.S. were acquired from the Homeland Infrastructure Foundation-Level Data (HIFLD) Open Data interface [17]. The dataset was filtered to include only open/active pharmacies, resulting in 62,946 pharmacies. We used information on the latitude and longitude of the 62,946 pharmacies to visualize their locations in ArcMap using the "Display X.Y. Data" tool. We limited our analysis to currently open pharmacies in the contiguous U.S., resulting in 61,589 unique locations. MUAs, defined by the U.S. Department of Health and Human Services (HHS), updated in June 2020, were acquired as a shapefile from the ArcGIS Online website [18]. CDC-SVI was computed using U.S. Census data encompassing the following four themes: 1) socio-economic status (SES), 2) housing composition and disability, 3) minority status and language, and 4) housing and transportation [19]. All data sources used in this study are publicly available and anonymized through the provided references. The spatial distribution of pharmacies color coded based on major brands as well the locations of Medically Underserved Areas (MUAs) are shown in Fig 1.

### Exposures

#### MUA status of each census block group

To define the status of each census block group with regards to MUA, the “Intersect” tool in ArcMap (a tool that calculates the geometric intersection of any number of polygons) was applied to the census block groups and MUA layers to generate a new layer that provides the MUA portion of each census block group. The attribute table of this new layer was exported to Microsoft Excel to calculate the proportion of the area within each census block group located within a MUA using the pivot table (one to many relationship).

The proportion of the area within each census block group located within a MUA ranged from zero to 100%. A simple majority-based approach was undertaken to label a census block group with a MUA status; a threshold of 50% (majority) of the total area was then applied to the census block groups to separate MUA from non-MUA block groups. A sensitivity analysis using various thresholds ranging from 50–75% showed no major changes to the distribution of MUA versus Non-MUA block groups. Furthermore, the locations of the 61,589 pharmacies were overlapped with the MUAs using ArcMap’s “Spatial Join” tool to assign MUA status to each pharmacy. The area proportion rather than the proportion of the people in the census block groups was used because of the lack of information on the MUAs’ population distribution within the census block groups. Considering the census block group resolution with an average area and population of 36 km^2^ and 1,483 people, respectively, using a geometric centroid was justified.

### The Centers for Disease Control and Prevention (CDC) Social Vulnerability Index (SVI)

The CDC-SVI was calculated for each census block group based on the CDC’s guidelines [20]. In brief, 15 variables (see S1 Table) in the four aforementioned themes were compiled for all 215,836 census block groups. Descriptions on generating and applying SVI scores are captured in CDC/ATSDR Social Vulnerability Index (SVI) [20]. In addition to the actual SVI percentiles, each census block group was assigned an SVI status based on SVI quartiles (i.e., Q1, Q2, Q3, and Q4 SVI). Higher SVI scores represent greater vulnerability of the community to negative effects caused by external stresses on human health [20].

### SVI-MUA

Another stratification of the census block groups was conducted by combining the SVI and MUA statuses using logical statements and VLOOKUP function in Excel (a function that could find a value associated with a common ID in two tables), generating eight subgroupings (e.g., Q1 SVI-MUA, Q1 SVI-non-MUA, Q2 SVI-MUA, Q2 SVI-non-MUA, Q3 SVI-MUA, Q3 SVI-non-MUA, Q4 SVI-MUA, and Q4 SVI-non-MUA. This subgrouping served as the primary exposure variable in this analysis.

### Pharmacy closure scenarios

This study examined the expected effects of pharmacy closures, regardless of brand pharmacy type, by modeling the expected additional travel distance from the centroid of each census block if the nearest pharmacy to a household was closed and the economic ramifications of this increase. The assumption is that the customers of a closed pharmacy will travel to the second nearest pharmacy. Because it is unlikely that the nearest pharmacies in all census block groups are closed simultaneously, individual block groups or the average/median of expected effects (additional costs in this study) were used instead of the sum across the U.S.

### Outcomes of interests

#### Travel distance to the nearest pharmacies from the centroid of each census block

The average travel distance to the two nearest pharmacies was calculated. The difference between these two was used to estimate the additional travel distance to the second pharmacy in the event of the closure of the nearest pharmacy. The following steps were taken to calculate the average travel distance: 1) the "Feature to Point" tool (this tool calculates the center of gravity of a polygon) in ArcMap Desktop 10.8.1 was used to convert the block group polygons into center points, 2) the "Generate Near Table" in ArcMap, with the "Maximum Number of Closest Matches" set to two, and “Planar” method (planar and Euclidean methods are the same and were used interchangeably in this work) was applied by using the centroids and pharmacies shapefiles as the input and near features, respectively, 3) the results were imported as text file and then loaded in Excel, 4) VLOOKUP function was used in Excel to create a master distance table using the block groups I.D.s as the common variable and finding their associated MUA and SVI status, population, households numbers, the distance to the two nearest pharmacies, and the name of each of these two pharmacies, and 5) pivot tables were created to generate summary statistics for various analyses purposes.

Specifically, we estimated travel distance using the planar/Euclidean method. While other studies have assessed population inequities using travel time [20, 21] and real street networks [22], our preference for the planar/Euclidean method is based on its ability to overcome the drawbacks of other approaches. The real street network relies on actual travel time, which can be complicated, considering that travel time varies depending on the time of the day when travel occurs, and the vehicle chosen for travel. In addition, using commercial products, such as Google Maps Distance Matrix API, to estimate the travel distance/time based on the real street network would be very expensive considering the number of census block groups (215,836) and pharmacies (61,589) this study examined. Finally, considering the small size of the census block groups the difference between the geodesic (a 3-D method that considers topography) and the planar (a 2-D method that calculates the distance between two points by calculating the length of a straight line between them) is neglectable. For these reasons, our models are based on the planar/Euclidean method.

However, we included a validation analysis that compares the planar/Euclidean method to the real street network method of estimating travel distance. After calculating the distances in ArcMap, we randomly sampled census block groups for various percentiles of distances (5%-95% at 5% intervals, 99%, 99.9%, and maximum distance). Google Maps was then used to calculate the actual street distance (the distances needed to be traveled using the suggested path with certain streets) among the selected latitude and longitudes of the centroid of the block groups and their associated nearest pharmacies.

#### Additional travel distance associated with the closure of the nearest pharmacy to the centroid of each census block

The aforementioned master distance table was used to calculate the additional travel distance to the next pharmacy following the closure of the nearest one. The total additional travel distance for each census block group (total travel) was calculated using the following equation:

Totaltravel=2×additionaltraveldistance×35×n (Eq 1)

where additional travel distance was calculated by subtracting the travel distance required for people living in a census block group to the two nearest pharmacies as explained in the previous section, 2 is a factor to account for the round trip to the pharmacy, 35 is the average number of times that households visit pharmacies per year for various reasons [23], and n is the number of households within each block group. Histograms for various stratifications (e.g., MUAs, SVIs, and MUA-SVI) were used to compare the distribution of the additional travel distance. These histograms depict the spread of the additional distances compared to summary statistics snapshots.

#### Economic impacts of the additional travel distance associated with the closing of the pharmacy nearest to the centroid of each census block

Based on standard mileage rates provided by the Internal Revenue Service (IRS) in 2022, we estimated the cost of travel to be approximately 58.5 cents per mile [24]. We also accounted for the environmental cost of travel, based on the amount of carbon dioxide (CO_2_) emission per mile of driving (404 grams) reported by the Environmental Protection Agency (EPA) in 2018 and the $50 social costs of CO_2_ emission (per metric ton), announced by the Environmental Defense Fund (EDF) [25, 26]. The social cost is a metric designed to estimate the economic costs, or climate damage, of one additional ton of carbon dioxide emission, and many researchers have commonly used this as the optimal CO_2_ price [16, 27]. Finally, we accounted for the lost time associated with the additional travel distance to the next pharmacy following the closure of the nearest one, using conservative earnings data by multiplying the additional travel time by hourly labor rates. The total additional travel time was estimated by dividing the total additional travel distance by 30 miles per hour (an estimation of the car speed within urban areas). We used the “Per Capita Income” variable in the census data and converted the values into hourly rates by dividing the income by 52 weeks and 40 hours per week, assuming full-time workers. Although the unemployment rate is significantly higher (p-value<<0.01) in census block groups in MUAs (average = 7.7%) compared to non-MUA ones (average = 5.4%), the actual percentages are very close. Thus, in this study, we assumed that the difference in the unemployment rates would not change our results.

## Results and discussion

### Census block groups characteristics

**Fig 2:**
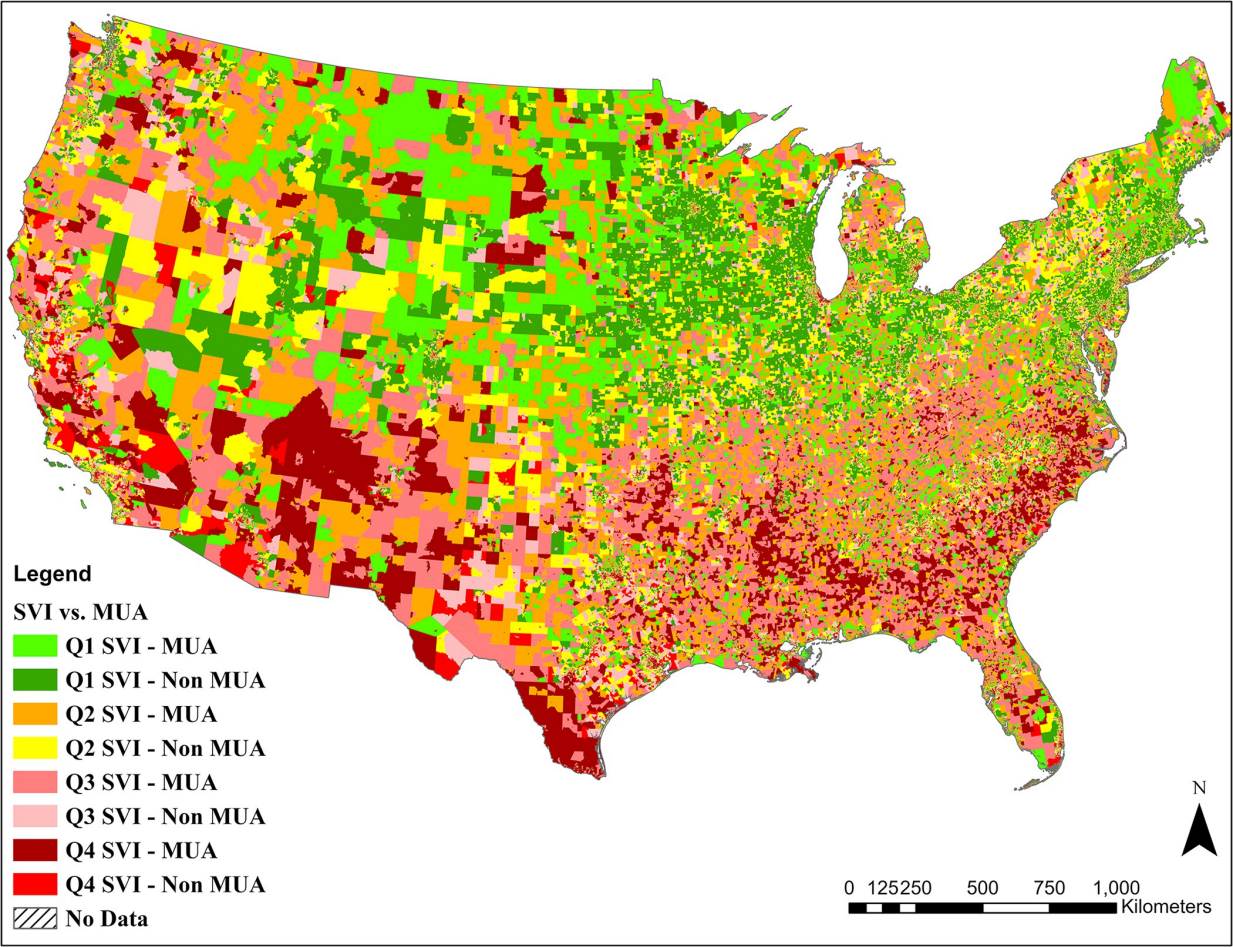
Stratification of the census block groups (n = 215,836) by combining the SVI (four quarters) and MUA (binary) statuses which led to eight subgroups.

Of the 215,836 census block groups within the contiguous U.S., 83,547 (38.71%) lie within MUAs. Fig 2 shows the spatial distribution of the eight SVI-MUA subgroups. Most Q4 SVI-MUA block groups, which include the ones with the highest levels of the vulnerable population living in medically underserved areas, are located in southern states, especially in Texas, Arizona, New Mexico, Alabama, and North and South Carolina, and some scattered areas within metropolitan areas such as Atlanta, Houston, Phoenix, Los Angeles, and New York.

The distribution of population in census block groups located in MUAs and Non-MUAs showed almost identical results. Even changing the MUA selection threshold to 100% did cause a significant difference between the population distribution of MUA and Non-MUA census block groups. This is an important finding as it shows that lower allocation of pharmacies in MUAs is not associated with smaller population sizes. S1 Fig shows the population distribution in census block groups in MUAs and Non-MUAs. Conversely, most Q1 SVI-non-MUA block groups, which include the ones with the lowest vulnerable population living in non-medically underserved areas, are primarily located in Midwest and North-Eastern states, mostly rural areas with low population and pharmacy densities. As shown in S2 Table, Q1 SVI -non-MUA with a population of 63,486,781 (19.79% of the U.S. population) and Q1 SVI–MUA with a population of 16,184,908 (5.05% of the U.S. population) are the classes with the highest and lowest population, respectively.

S2 Table shows the population breakdown within each subgroup. Compared to their non-MUA counterparts, areas with the highest social vulnerability indices (Q4 SVI) were over-represented in MUAs, with a mean of 0.435±0.001 and 0.602±0.001, respectively. Such a substantial difference suggests that people with the greatest vulnerability also reside in MUAs.

### Estimated travel distance to the nearest pharmacies from the centroid of each census block

**Fig 3:**
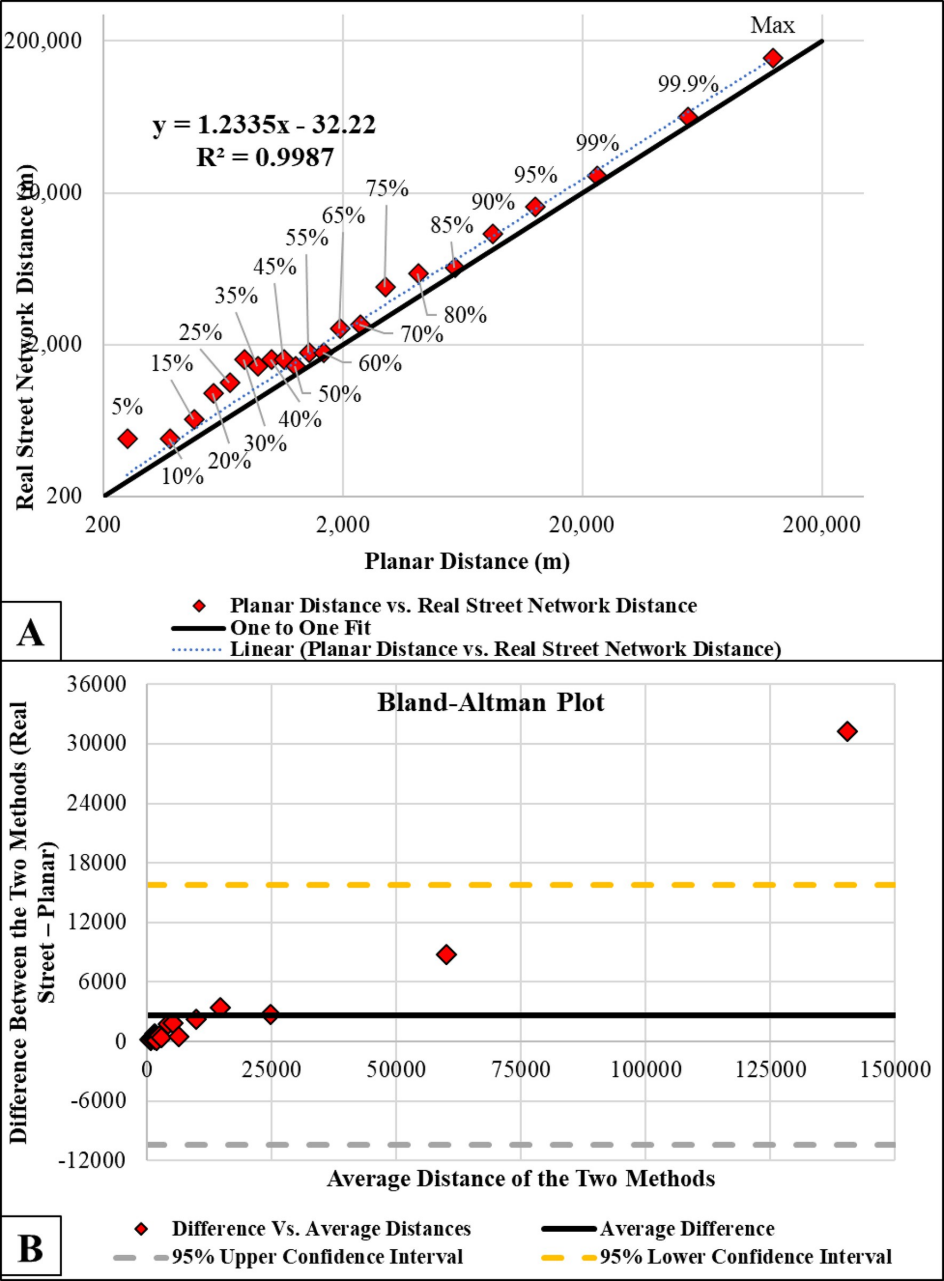
Distance between the center of census block groups and the nearest pharmacies (planar Euclidean distance calculated in ArcMap) and real street network (calculated using Google Map) at various distance percentiles, and B) Bland-Altman plot showing the difference and average distances of the planner Euclidean and real street network methods.

Fig 3 shows a comparison of the planar/Euclidean method and the real street network method of estimating distance. We observed a statistically significant correlation (p-value<0.01, R^2^ = 0.999) between the planar Euclidean distance calculated in ArcMap and real street network (calculated using Google Map) methods at various percentiles of distances. This significant correlation suggests that estimates using both methods are very similar, justifying and validating the use of the planar method for various ranges of distances. However, using the Bland-Altman plot as shown in Fig 3B, we found that the planar method always underestimated the distances compared to the real-world method (all points are above or below the zero line), making our estimation more conservative. The discrepancy between the two methods was generally worse for distances above 25,000 m than those below 25,000 m.

Table 1 shows the average distances to the nearest pharmacy, additional travel distances, and the percentage of changes in travel distances following pharmacy closure for both the nearest and second nearest pharmacies inside and outside MUAs. Our results suggest that currently, residents of MUA have almost two times greater travel distances to pharmacies than non-MUAs (4,269 m (2.65 miles) vs. 2,388 m (1.48 miles)), and this disparity is exaggerated with pharmacy closures (107% increase in travel distance in MUAs vs. 75% increase in travel distance in non-MUAs.

**Table 1:** The average distances to the nearest pharmacy, additional travel distances, and the percentage following pharmacy closure for the nearest and second nearest pharmacies inside and outside MUAs.

Scenario	MUA		
	Distance (m)	Additional Travel Distance (m)	Additional Travel Distance (%)
The nearest pharmacy to the center of the census block groups	4,269±46* (2.65±0.03 mi)	1,714±33 (1.06±0.02 mi)	107.2%±6.2%
The second nearest pharmacy to the center of the census block groups	5,989±61** (3.72±0.04 mi)	N/A	N/A
Scenario	Non-MUA		
	Distance (m)	Additional Travel Distance (m)	Additional Travel Distance (%)
The nearest pharmacy to the center of the census block groups	2,388±20 (1.48±0.01 miles)	862±14	75.0%±1.8%
The second nearest pharmacy to the center of the census block groups	3,255±27 (2.02±0.02 miles)	N/A	N/A

* The value after ± represents the variation in mean estimation required to build 95% confidence intervals.

** the sum of average distance and average additional distance are not precisely equal to the average distance to the second nearest pharmacy to the center of census block groups because some census block groups were excluded from the calculation since there was no second pharmacy within the search radius for them.

**Fig 4:**
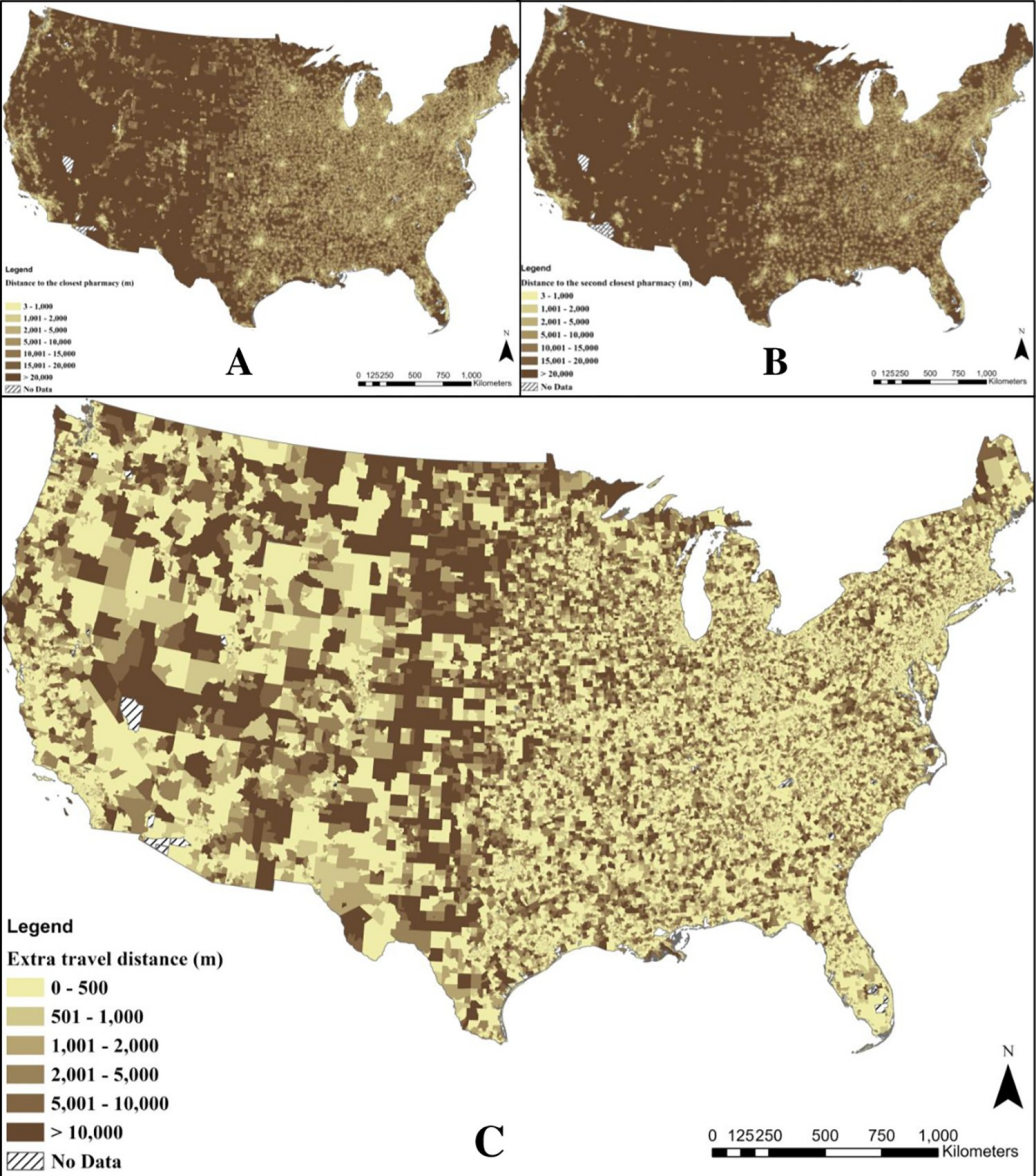
Spatial distribution of the distance from the centroid of census block groups (n = 215,836) to the nearest pharmacy B) the spatial distribution of the distance from the centroid of census block groups to the second nearest pharmacy and C) the spatial distribution of additional travel distance following the closure of the nearest pharmacy.

Fig 4 shows the spatial distribution of the distance from the centroid of census block groups to the first and second nearest pharmacies in the contiguous U.S. Analysis of variance tests revealed significant differences in travel distances across all 8 SVI-MUA subgroups.

### Estimated additional travel distance associated with the closing of the pharmacy nearest to the centroid of each census block

Fig 4C shows the additional travel distance caused by the hypothetical closure of the nearest pharmacy. The highest differences (up to 124,713 m additional travel distance) were observed across a hypothetical line drawn in the middle of the U.S. to separate the West from the East, as shown in Fig 4C. This finding, combined with the discoveries mentioned above on additional distances, shows that people living in rural areas are more likely to travel a higher distance to reach the second nearest pharmacy following the closure of the nearest one than people living in urban areas.

**Fig 5:**
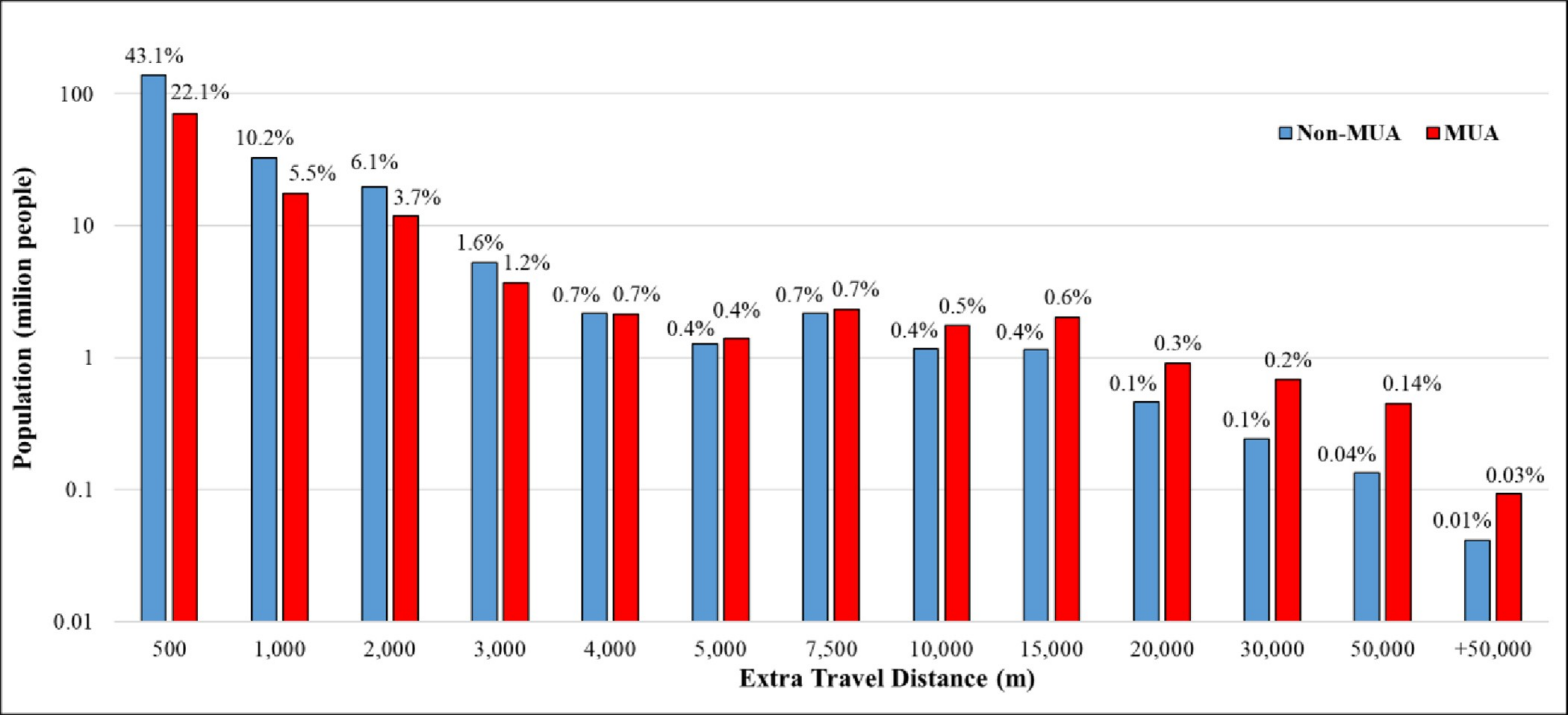
Distribution of population living in census block groups and additional travel distance following the closure of the nearest pharmacy.

Fig 5 provides a population view of additional travel distance by MUA vs. non-MUA. While most of the U.S. population (138,265,700 in MUAs and 70,750,713 in non-MUAs) will face an additional travel distance of less than 500 m, 9,101,035 people (3,203,723 non-MUA plus 5,897,312 MUA) are living in areas in which a pharmacy closure will result in an additional travel distance of more than 10,000 m (6.2 miles), on average, to reach to the next open pharmacy.

### Estimated economic impacts due to additional travel distance associated with the closing of the pharmacy nearest to the centroid of each census block

Table 2 shows the average annual economic costs due to additional travel (car mileage, CO_2_ emission, and lost hours labor cost) and the average cost per additional km of travel for each studied group. The economic losses were $34,834 ± $668 and $22,720 ± $326 for a census block group within MUAs, and non-MUAs, respectively. The breakdown for the three loss categories (car mileage, CO_2_ emission, and lost hours labor cost) were $11,576 ± $175, $400 ± $6, and $10,744 ± $150 in non-MUAs and $19,970 ± $380, $690 ± $13, and $14,174 ± $283 in MUAs, respectively. The average additional costs for one kilometer of additional travel are shown in Table 2. These values, on average, were significantly different (p-value<<0.01), with slightly higher average costs in census block groups located in non-MUAs ($5.94 ± $0.00) compared to MUAs ($5.77 ± $0.00).

**Table 2:** Average annual economic costs breakdown due to additional travel within various studied groups.

Group	# of block groups	Average total distance (km)*	Car mileage	CO_2_ emission	Lost hours labor	Total	Average cost per additional km of travel
Q1 SVI–MUA	11,849	59,430±2,502**	$21,602±909	$745±31	$20,447±867	$42,796±1791	$5.74±$0.01
Q1 SVI—Non-MUA	42,110	34,432±774	$12,516±281	$432±9	$14,054±289	$27,003±571	$5.88±$0.01
Q2 SVI–MUA	17,501	74,582±2,745	$27,110±997	$936±34	$21,219±794	$49,266±1809	$5.73±$0.01
Q2 SVI—Non-MUA	36,458	35,191±985	$12,792±357	$441±12	$12,025±311	$25,259±673	$5.93±$0.01
Q3 SVI–MUA	23,018	60,443±2,084	$21,971±757	$758±26	$14,802±536	$37,532±1308	$5.74±$0.01
Q3 SVI—Non-MUA	30,941	30,917±1,086	$11,238±394	$388±13	$8,839±290	$20,466±693	$5.97±$0.01
Q4 SVI–MUA	31,179	38,141±1,474	$13,864±535	$478±18	$7,373±319	$21,716±861	$5.84±$0.01
Q4 SVI—Non-MUA	22,780	22,979±1,052	$8,352±382	$288±13	$5,163±246	$13,804±634	$6.03±$0.01
MUA	83,547	54,938±833	$19,970±380	$690±13	$14,174±283	$34,834±668	$5.77±$0.00
Non-MUA	132,289	31,847±606	$11,576±175	$400±6	$10,744±150	$22,720±326	$5.94±$0.00
Total	215,836	40,787±504	$14,825±564	$512±19	$12,072±143	$27,409±328	$5.88±$0.00

* Values are calculated based on Eq 1 with proper unit conversion.

** The value after ± represents the variation in mean estimation required to build 95% confidence intervals.

**Fig 6:**
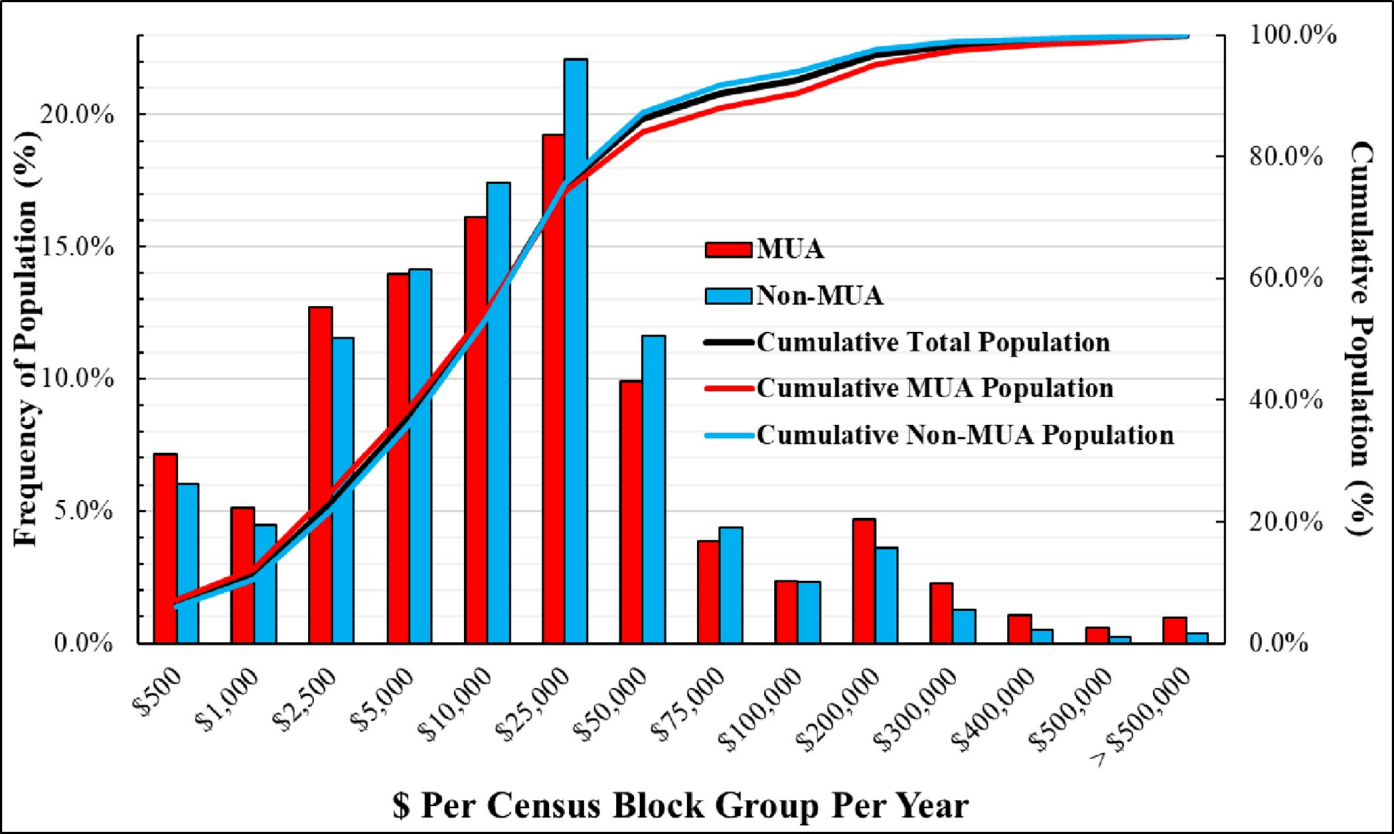
Distribution of population living in census block groups and economic losses associated with additional travel distances.

Table 2 also shows the breakdown of the eight SVI-MUA and three economic loss groups. To put these numbers in perspective, a census block group in the U.S. has an average of 550 households with an average population of 1,483. While the average dollar amount might seem low, the maximum numbers could be as high as $3,115,102 and $3,140,083 for block groups in MUAs vs. Non-MUAs. The associated resident populations for MUA vs. Non-MUA census block groups (with the maximum numbers) are 1,454 and 1,456 people, respectively, which means an additional average cost of ~$2150 per person per year due to a potential pharmacy closure within those block groups. Moreover, geospatial and statistical analyses revealed that 1,842,672 people are estimated to live in census block groups where a pharmacy closure could cause more than $500,000 annual loss (please refer to S3 Table). Fig 6 shows the histograms for MUA and Non-MUA areas that represent the number of people with each economic loss interval. In S3 Table, the cost intervals represent the additional annual costs attributed to the entire population collectively in the census block.

In this spatial-economic analysis of the differential impact of planned pharmacy closures on medically underserved and socially vulnerable areas in the US, our findings reveal that residents of MUAs will have almost two times greater travel distances to pharmacies than non-MUAs. Individuals residing in MUAs will experience significantly greater annual economic costs than non-MUAs, estimated at $12,000 excess of costs in non-MUAs.

The findings of this research align well and add to the previous research on the effect of pharmacy closure on the population. Although the total number of US pharmacies increased by 7.8% from 2009 to 2015, 9,564 (12.8%) pharmacies closed during the same period [5]. Guadamuz *et al*. (2020) found that the risk of pharmacy closure was significantly higher in urban areas serving low-income, uninsured, and publicly uninsured individuals [5]. Another recent study highlighted social determinants of pharmacy deserts and showed that pharmacy deserts tend to have higher populations of residents below the federal poverty line, lack health insurance, and reside in areas of health professional shortages [11]. Geographic accessibility to pharmacies is pivotal to medication access, especially for the elderly, lower SES, and historically excluded people of color, and studies have shown that pharmacy closures can contribute to poorer medication adherence among chronically ill patients [28, 29]. Thus, the additional financial and time cost for individuals, particularly those living in rural communities and urban pharmacy deserts, could limit medication access and affect overall medication utilization, adherence, and related health outcomes.

These findings have significant policy implications. Surprisingly, it is not difficult to close a retail or hospital pharmacy. Even though pharmacies are regulated at the local, state, and federal levels, there are no federal requirements for closing a pharmacy. Pharmacies must provide a public announcement, return their license, handle remaining substances appropriately, and store records and prescription files appropriately to close [30]. None of these regulations are problematic for big chain retail pharmacies where any remaining substances can be transferred to the other pharmacies within the chain, and the records storage will be unaffected because the chain is still in business. These factors make it relatively easier for big retail or hospital pharmacies to close because they can bypass many hurdles that small independent pharmacies may face. Independent pharmacies account for more than 30% of all retail pharmacies and are often located in medically underserved and rural communities [31]. Rural residents may have special relationships with independent pharmacies that may not exist with retail chain pharmacies. Consequently, the closure of independent pharmacies creates meaningful disruptions to pharmacy access in medically underserved and rural communities. Comparatively, hospitals face stiffer regulations to close. For example, according to §1866(b) of the Social Security Act, health provider services such as hospitals may voluntarily cease their operation by notifying the State survey Agency (S.A.) via written notice of their desire. Subsequently, the S.A. informs the Medicare Administrative Contractor (MAC). After MAC agrees with the termination date, a public announcement of the exact closure date is posted on the Centers for Medicare & Medicaid Services (CMS) website [32]. Our findings suggest the need for additional regulations at the State and Federal levels to ensure populations are not disproportionately affected. It is also imperative that pharmacies have significant interactions and bidirectional discourse with community stakeholders before pharmacy closure decisions are made.

Consideration should be given to expanding alternative medication delivery services like mail-order pharmacies to mitigate the impact of these pharmacy closures. To mitigate the impact of these pharmacy closures, mail-order pharmacies operate solely through a health insurer’s pharmacy benefit management. Since COVID-19, this approach has gained momentum to mitigate access and transportation barriers by mailing prescription medications to a patient’s home [33]. Others have reported that the utilization of any mail-order prescription increased from 6.9% to 10.3% between 1996 and 2018, representing a 4.86% rise over the baseline [33]. These significant increases in mail-order pharmacy correlate with a decrease in in-person pharmacy services and align with the business reasons for impending pharmacy closures [33]. Such a shift could decrease or even eliminate travel time to the nearest pharmacy while also contributing to improving health outcomes. In fact, Schmittdiel *et al*. (2011) showed that using mail-order pharmacies was positively associated with increased medication adherence [34]. However, It is important to note that immunizations and COVID testing are either not feasible or challenging if a mail-order pharmacy becomes the only option. While mail-order pharmacies can improve medication access, they do not address the more intangible aspects of interactions with a brick-and-mortar pharmacy where, for example, patients may obtain medication counseling or health advice. Other options such as online pharmacies are also on the increase, offering home delivery; however, some online pharmacies require no valid prescription from a doctor, lack access to a U.S. state-licensed pharmacist and often lack legitimacy and monitoring [35].

Other potential solutions should explore, leverage and strengthen innovative partnerships that could allow the presence of pharmacies in unconventional places. For example, intra-industry partnerships allow pharmacy brands, like CVS and Walgreens, to partner in MUAs, pooling resources to cater to underserved populations. Intra-industry collaboration exists; however, rarely does it occur, primarily for community benefit. For instance, pharmaceutical giants Pfizer and BioNTech joined forces on more than one occasion to develop new drugs, most recently for creating a novel mRNA shingles vaccine [36]. Using Pfizer’s scientists and BioNTech’s patented mRNA technology, the two companies aimed to create a first-of-its-kind mRNA-based shingles vaccine. The pair worked together in a similar capacity to develop a widely distributed vaccine for COVID. Inter-industry partnerships, including multi-sectoral partnerships, may also identify opportunities to translate previous inter-industry models to address pharmacy access. For example, the Food as Medicine collaborative fosters an inter-industry partnership that bridges healthcare and food systems to address food insecurity and improve population health simultaneously [37]. Integrated service delivery models such as medical homes and accountable care organizations (ACOs) foster multi-sectoral participation because of their ability to engage multiple healthcare system stakeholders to improve quality and contain costs. These partnerships may improve pharmacy access, streamline processes, spark innovations, and improve patient outcomes.

This study is not without limitations. We only include community retail pharmacies in this analysis. There are other pharmacies, for example, those located inside hospitals (representing less than 1% of all pharmacies), which are not captured in our analysis. Although most households within MUAs have access to personal cars (~87.8%), the percentages of households without a personal vehicle in census block groups located in MUAs are significantly higher compared to the ones located in Non-MUAs (p-value<<0.01 with averages of 12.2% and 7.2%, respectively). Thus, another limitation of this work is that some individuals and families do not own their vehicles and must rely on public transportation to get from place to place. This limitation, in addition to the use of a planar/Euclidean methodology to estimate the distances, underscores the conservative nature of this analysis. This is mostly because individuals who rely on public transportation might experience additional travel distances than those reported in this study. Thirdly, the reported CO_2_ emission costs appear to reflect the US position on climate change (up until August 2022, when the Inflation Reduction Act, which provides $369 billion for climate and clean energy provisions, was passed in Congress), representing a shadow price associated with meeting a specific emission target. Fourthly, this study did not consider uncertainties associated with estimating the factors mentioned above. Finally, another externality associated with additional travel distance is the likelihood of car accidents, which this study does not estimate.

## Conclusion

Compared to those residing in non-MUAs, persons residing in MUAs already have poorer access to pharmacies, as evidenced by greater travel times. As this study shows, the hypothetical closure of the nearest pharmacy will disproportionately affect those living in MUAs and exacerbate disparities between MUA and non-MUA residents. Notably, additional travel distance contributes to other serious environmental challenges, such as air and noise pollution and the increased threat of global climate change, which this study does not capture. Other air pollutants such as nitrogen dioxide (NO_2_) and methane (CH_4_) could also be considered when estimating the impacts of additional travel distance. Beyond losing access to a health professional in the vicinity, pharmacy closures, especially in MUAs, also may harm the overall socio-economic status of the area as pharmacies often employ its community’s residents. Some pharmacies also sell groceries in many communities in food deserts, so closing these pharmacies can potentially exacerbate food insecurity. Finally, when patients switch to the next pharmacy following the closure of the nearest pharmacy, this switch may overwhelm the remaining pharmacies or hinder pharmacists from allotting enough time to see patients. Being overwhelmed or overburdened is associated with prescription errors [38, 39]. Moreover, the enhanced geographic barriers to pharmacies caused by pharmacy closures can negatively impact patients’ adherence to pharmacological therapy and complex drug regimens [40, 41]. Poor medication utilization and adherence increase the likelihood of patient mortality and the misuse of other medical services, such as hospitalizations and emergency department visits [42]. Annually, medication nonadherence costs US$100 to U$290 billion, which contributes to the waste of U.S. healthcare spending [42]. Future work should examine the probability of pharmacy closures by retrospectively assessing factors contributing to closure risk.

## Supporting information

S1 Table List of 15 variables used to calculate the Centers for Disease Control and Prevention (CDC) Social Vulnerability Index (SVI). (DOCX)

S2 Table The breakdown of the population living within each of the eight SVI-MUA groups. (DOCX)

S3 Table The population living in census block groups with various additional annual costs due to the closure of the nearest pharmacy. (DOCX)

S1 Fig Population distribution in census block groups in MUAs with 50% threshold (n = 83,547), 100% threshold (n = 32,294), and Non-MUAs (n = 132,289). (DOCX)
