# Supplementary material for: Rethinking access to care: A spatial-economic analysis of the potential impact of pharmacy closures in the United States
Source: PLoS One. 2023 Jul 27;18(7):e0289284. doi: 10.1371/journal.pone.0289284 (PMC10374066; doi:10.1371/journal.pone.0289284)
Supplement: S1 Table — (DOCX) [file pone.0289284.s001.docx]

**S1 Table.** List of 15 variables used to calculate the Centers for Disease Control and Prevention (CDC) Social Vulnerability Index (SVI)

| **Theme** | **Variable** |
| --- | --- |
| **1** | Persons below poverty estimate |
|  | Civilian (age 16+) unemployed |
|  | Per capita income |
|  | Persons (age 25+) with no high school diploma |
| **2** | Persons aged 65 and older |
|  | Persons aged 17 and younger |
|  | Civilian noninstitutionalized population with a disability |
|  | Single parent household with children under 18 |
| **3** | Minority (all persons except white, non-Hispanic) |
|  | Persons (age 5+) who speak English "less than well" |
| **4** | Housing in structures with 10 or more units estimate |
|  | Mobile homes estimate |
|  | At household level (occupied housing units), more people than rooms estimate |
|  | Households with no vehicle available |
|  | Persons in institutionalized group quarters |
