# Supplementary material for: Rethinking access to care: A spatial-economic analysis of the potential impact of pharmacy closures in the United States
Source: PLoS One. 2023 Jul 27;18(7):e0289284. doi: 10.1371/journal.pone.0289284 (PMC10374066; doi:10.1371/journal.pone.0289284)
Supplement: S2 Table — (DOCX) [file pone.0289284.s002.docx]

**S2 Table.** The breakdown of the population living within each of the eight SVI-MUA groups.

| **SVI-MUA Classification** | | |
| --- | --- | --- |
| Class | Population | Population (%) |
| Q1 SVI - MUA | 16,184,908 | 5.05% |
| Q1 SVI - Non-MUA | 63,486,781 | 19.79% |
| Q2 SVI - MUA | 24,851,512 | 7.75% |
| Q2 SVI - Non-MUA | 56,862,543 | 17.73% |
| Q3 SVI - MUA | 31,817,970 | 9.92% |
| Q3 SVI - Non-MUA | 48,439,468 | 15.10% |
| Q4 SVI - MUA | 42,960,301 | 13.39% |
| Q4 SVI - Non-MUA | 36,138,935 | 11.27% |
| **SVI Classification** | | |
| Class | Population | Population (%) |
| Q1 | 79,671,689 | 24.84% |
| Q2 | 81,714,055 | 25.48% |
| Q3 | 80,257,438 | 25.02% |
| Q4 | 79,099,236 | 24.66% |
| **MUA Classification** | | |
| Non-MUA | 204,927,727 | 63.89% |
| MUA | 115,814,691 | 36.11% |
| **Total Population** | | 320,742,418 |
