# Supplementary material for: Rethinking access to care: A spatial-economic analysis of the potential impact of pharmacy closures in the United States
Source: PLoS One. 2023 Jul 27;18(7):e0289284. doi: 10.1371/journal.pone.0289284 (PMC10374066; doi:10.1371/journal.pone.0289284)
Supplement: S3 Table — (DOCX) [file pone.0289284.s003.docx]

**S3 Table.** The population living in census block groups with various extra annual cost due to the closure of the nearest pharmacy. the cost intervals represent the annual extra cost due to the closure of the nearest pharmacy that is attributed to the entire population, collectively in the census block.

| **Annual Cost Interval** | **Population** | | | **Frequency (%)** | | |
| --- | --- | --- | --- | --- | --- | --- |
|  | **All** | **MUA** | **Non-MUA** | **All** | **MUA** | **Non-MUA** |
| < $500 | 20,611,821 | 8,277,948 | 12,333,873 | 6.4% | 7.1% | 6.0% |
| $500 - $1,000 | 15,111,936 | 5,915,169 | 9,196,767 | 4.7% | 5.1% | 4.5% |
| $1,000 - $2,500 | 38,364,059 | 14,706,778 | 23,657,281 | 12.0% | 12.7% | 11.5% |
| $2,500 - $5,000 | 45,163,512 | 16,199,865 | 28,963,647 | 14.1% | 14.0% | 14.1% |
| $5,000 - $10,000 | 54,379,375 | 18,660,063 | 35,719,312 | 17.0% | 16.1% | 17.4% |
| $10,000 - $25,000 | 67,570,579 | 22,280,256 | 45,290,323 | 21.1% | 19.2% | 22.1% |
| $25,000 - $50,000 | 35,341,389 | 11,479,305 | 23,862,084 | 11.0% | 9.9% | 11.6% |
| $50,000 - $75,000 | 13,484,041 | 4,494,483 | 8,989,558 | 4.2% | 3.9% | 4.4% |
| $75,000 - $100,000 | 7,432,324 | 2,722,961 | 4,709,363 | 2.3% | 2.4% | 2.3% |
| $100,000 - $200,000 | 12,859,217 | 5,442,745 | 7,416,472 | 4.0% | 4.7% | 3.6% |
| $200,000 - $300,000 | 5,193,877 | 2,625,854 | 2,568,023 | 1.6% | 2.3% | 1.3% |
| $300,000 - $400,000 | 2,235,940 | 1,204,915 | 1,031,025 | 0.7% | 1.0% | 0.5% |
| $400,000 - $500,000 | 1,151,676 | 679,078 | 472,598 | 0.4% | 0.6% | 0.2% |
| > $500,000 | 1,842,672 | 1,125,271 | 717,401 | 0.6% | 1.0% | 0.4% |
| Total | 320,742,418 | 115,814,691 | 204,927,727 | 100.0% | 100.0% | 100.0% |
