# Supplementary material for: Rethinking access to care: A spatial-economic analysis of the potential impact of pharmacy closures in the United States
Source: PLoS One. 2023 Jul 27;18(7):e0289284. doi: 10.1371/journal.pone.0289284 (PMC10374066; doi:10.1371/journal.pone.0289284)
Supplement: S1 Fig — (DOCX) [file pone.0289284.s004.docx]

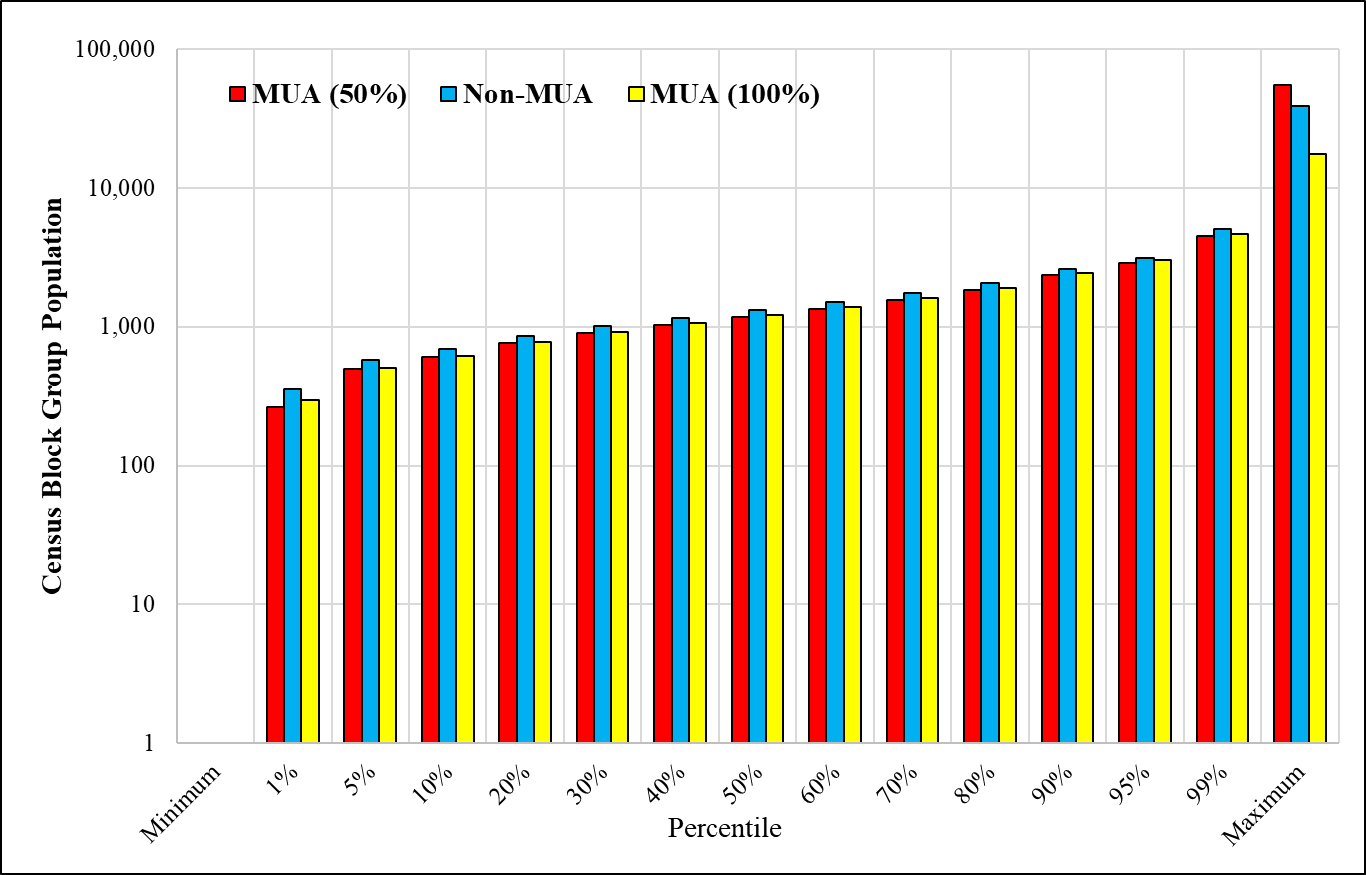


**S1 Fig.** Population distribution in census block groups in MUAs with 50% threshold (n=83,547), 100% threshold (n=32,294), and Non-MUAs (n=132,289)
